# Argininosuccinate lyase deficiency causes blood-brain barrier disruption via nitric oxide–mediated dysregulation of claudin expression

**DOI:** 10.1172/jci.insight.168475

**Published:** 2023-09-08

**Authors:** Jordan Kho, Urszula Polak, Ming-Ming Jiang, John D. Odom, Jill V. Hunter, Saima M. Ali, Lindsay C. Burrage, Sandesh C.S. Nagamani, Robia G. Pautler, Hannah P. Thompson, Akihiko Urayama, Zixue Jin, Brendan Lee

**Affiliations:** 1 Department of Molecular and Human Genetics and; 2 Department of Radiology, Baylor College of Medicine, Houston, Texas, USA.; 3 Texas Children’s Hospital, Houston, Texas, USA.; 4 Department of Molecular Physiology and Biophysics, Baylor College of Medicine, Houston, Texas, USA.; 5 Department of Neurology, University of Texas Health Science Center, Houston, Texas, USA.

**Keywords:** Genetics, Neuroscience, Endothelial cells, Neurological disorders, Nitric oxide

## Abstract

Nitric oxide (NO) is a critical signaling molecule that has been implicated in the pathogenesis of neurocognitive diseases. Both excessive and insufficient NO production have been linked to pathology. Previously, we have shown that argininosuccinate lyase deficiency (ASLD) is a novel model system to investigate cell-autonomous, nitric oxide synthase–dependent NO deficiency. Humans with ASLD are at increased risk for developing hyperammonemia due to a block in ureagenesis. However, natural history studies have shown that individuals with ASLD have multisystem disease including neurocognitive deficits that can be independent of ammonia. Here, using ASLD as a model of NO deficiency, we investigated the effects of NO on brain endothelial cells in vitro and the blood-brain barrier (BBB) in vivo. Knockdown of *ASL* in human brain microvascular endothelial cells (HBMECs) led to decreased transendothelial electrical resistance, indicative of increased cell permeability. Mechanistically, treatment with an NO donor or inhibition of *Claudin-1* improved barrier integrity in ASL-deficient HBMECs. Furthermore, in vivo assessment of a hypomorphic mouse model of ASLD showed increased BBB leakage, which was partially rescued by NO supplementation. Our results suggest that ASL-mediated NO synthesis is required for proper maintenance of brain microvascular endothelial cell functions as well as BBB integrity.

## Graphical abstract

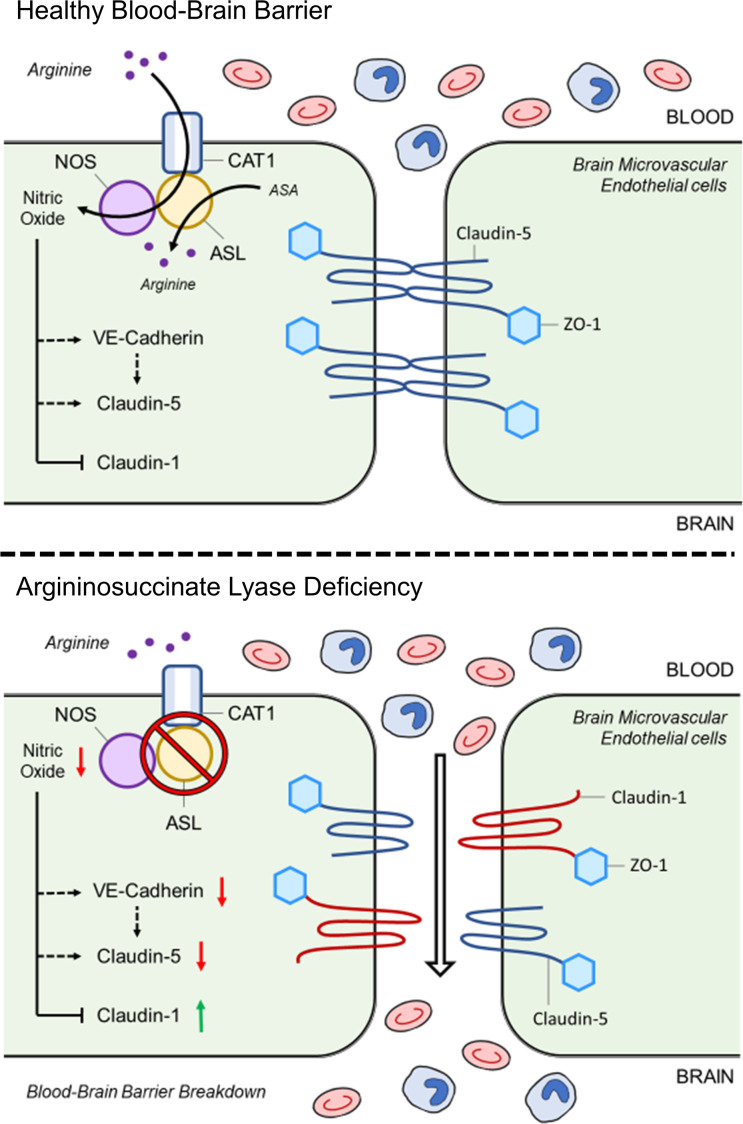

## Introduction

Argininosuccinate lyase (ASL), which catalyzes the fourth reaction in the urea cycle, cleaves argininosuccinic acid into arginine and fumarate. Biallelic pathogenic variants in *ASL* cause ASL deficiency (ASLD; argininosuccinic aciduria; OMIM #207900), the second most common urea cycle disorder (UCD), which has an estimated incidence of 1 in 218,750 births (1). ASLD is often characterized by an excessive accumulation of ammonia (hyperammonemia) in blood, a feature that is shared by the majority of UCDs. In addition to hyperammonemia, individuals with ASLD can have additional phenotypes, including hypertension, liver disease, and neurological dysfunction (2). A United Kingdom–wide study of 56 individuals with ASLD showed that neurological dysfunction is the most common complication in ASLD (3). Ammonia is a neurotoxin, and thus, hyperammonemia is a well-studied cause for neurological dysfunction in UCDs. Previous studies have shown that age at disease onset, peak ammonia levels, and duration of hyperammonemia are important predictors of neurological consequences in individuals with UCD (4, 5). As compared with UCDs with a more proximal block in ureagenesis, such as ornithine transcarbamylase deficiency or carbamoyl-phosphate deficiency, individuals with ASLD have fewer episodes of acute hyperammonemic crises (6). Furthermore, plasma ammonia or argininosuccinic acid (ASA) levels have not always correlated with neurological outcomes, indicating that hyperammonemia and ASA levels may not be the sole dominant factors driving the neurological phenotype in individuals with ASLD (3, 7).

In addition to playing an essential role in the urea cycle in the liver, ASL is expressed ubiquitously in multiple tissues; one potential role for ASL expression in the tissues is endogenous l-arginine and nitric oxide (NO) synthesis as part of the NO/citrulline cycle. We have previously discovered that ASL is required not only for the synthesis of arginine, the substrate for nitric oxide synthase (NOS), via recycling of citrulline, but also for channeling extracellular arginine to NOS (8). Because of this intracellular metabolite compartmentation, loss of ASL leads to cell-autonomous deficiency of NO production from intracellular citrulline/arginine and extracellular arginine. NO is a signaling molecule that plays important roles in neuronal function, differentiation, and survival (9, 10). Studies from our and other groups have suggested that the neurological outcome in individuals with ASLD may be caused by impaired NO production. In one study, we showed that NO therapy in an individual with ASLD was associated with improvements in certain neuropsychological outcome measures (11). Lerner et al. showed that conditional deletion of *Asl* in the locus coeruleus of mice resulted in reduced neuronal tyrosine hydroxylase activity and catecholamine synthesis, leading to impaired response to stressful stimuli and increased seizure reactivity. Interestingly, they showed that NO donors could normalize catecholamine production and rescue the phenotypes (12). A more recent study showed that mice with loss of ASL in ALDH1A1^+^ dopaminergic neurons in the substantia nigra pars compacta had motor and cognitive deficits (13) and that some deficits could also be ameliorated by NO supplementation (13).

The pathophysiology of neurocognitive dysfunction is complex and likely involves multiple cell types. Blood-brain barrier (BBB) dysfunction and breakdown have been associated with many neurological disorders, including cerebral ischemia, brain trauma, multiple sclerosis, brain tumors, and central nervous system (CNS) infections (14, 15). The BBB is formed by a monolayer of specialized endothelial cells lining the brain microvasculature. These human brain microvascular endothelial cells (HBMECs) are mechanically interconnected by tight junctions and act as a restrictive paracellular diffusion barrier (16). They are enveloped by pericytes and astrocytes, which regulate barrier induction and maintenance (17). NO has been shown to both decrease and increase BBB permeability, depending on the amount of NO, disease stages, and cellular contexts (18–22). Previously, we have shown that ASL is required for the synthesis of NO in endothelial cells and that endothelium-specific deletion of *Asl* in mice resulted in endothelial dysfunction, leading to hypertension (23). We also identified alterations in gene expression that are associated with BBB integrity, including claudin-5 (*CLDN5*), claudin-1 (*CLDN1*), and cadherin 5 (*CDH5*) (23). In the present study, we investigated the roles of ASL and NO signaling in regulating BBB permeability using HBMECs. Furthermore, we assessed the BBB in a hypomorphic mouse model of ASLD and examined the BBB from MRI results of 4 individuals with ASLD.

## Results

### siRNA-mediated knockdown of ASL in HBMECs.

**Figure 1:**
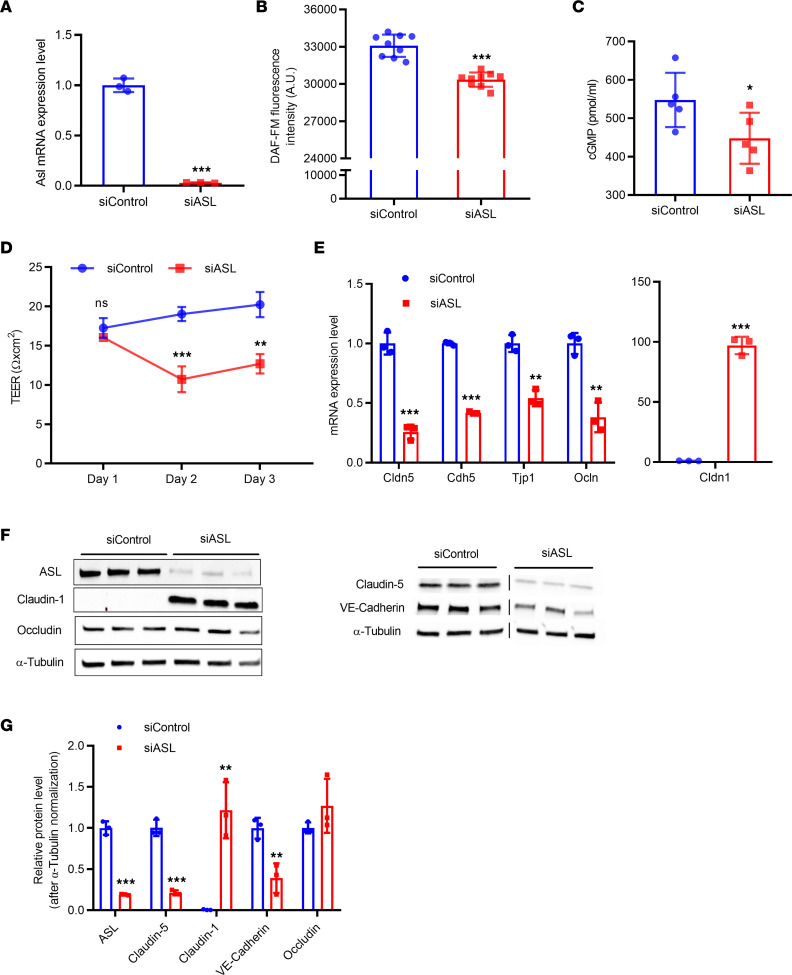
*ASL* knockdown in HBMECs leads to reduced NO signaling, decreased paracellular permeability, and dysregulated expression of BBB-associated genes. (**A**) Relative mRNA expression of ASL in HBMECs 2 days after transfection with control and ASL siRNAs (siControl and siASL) (*n* = 3). (**B**) Intracellular levels of NO in siControl and siASL-transfected HBMECs as measured by DAF-FM diacetate fluorescence assay (*n* = 9). (**C**) Intracellular cGMP levels in siControl and siASL-transfected HBMECs as measured by ELISA (*n* = 5). (**D**) Quantitative analysis of TEER level at 1–3 days after transfected HBMECs were seeded on Transwell inserts (*n* = 3). (**E**) Relative mRNA expression of *CLDN5*, *CDH5*, *TJP1*, *OCLN*, and *CLDN1* in HBMECs 2 days after siRNA-mediated knockdown (*n* = 3). (**F**) Western blot analysis of ASL, CLDN1, OCLN, CLDN5, and VE-Cadherin protein levels in HBMECs 2 days after siControl and siASL transfection. Samples were run on 2 separate gels, and α-Tubulin was used as the internal control (*n* = 3). (**G**) Protein abundance was quantitatively measured after normalization to α-Tubulin level. Bar graphs represent mean values while error bars represent the standard deviation. *P < 0.05, **P < 0.01, and ***P < 0.001. Student’s *t* test. A.U., arbitrary units.

We previously demonstrated that ASL is required for NO production in primary human aortic endothelial cells (HAECs) (23). We also generated induced pluripotent stem cell–derived (iPSC-derived) endothelial cells (ECs) from individuals with and without ASLD. Compared with control cells derived from healthy individuals, iPSC-derived ECs generated from patients with ASLD demonstrated NO deficiency (23). To correlate these findings in HBMECs, we knocked down *ASL* expression in primary HBMECs using siRNA-mediated gene silencing. Two days after siRNA knockdown, *ASL* expression was significantly downregulated (Figure 1A). We measured intracellular levels of NO in ASL-deficient HBMECs and found that consistent with HAECs and iPSC-derived ECs, *ASL* knockdown led to a reduction in NO production (Figure 1B). This was also accompanied by decreased intracellular cGMP, a downstream target of NO signaling (Figure 1C).

To assess how loss of ASL affects barrier properties, we seeded HBMECs onto Transwell inserts 24 hours after siRNA knockdown. Transendothelial electrical resistance (TEER), a marker of BBB permeability, was measured from 24 hours to 72 hours after subculture. We found that ASL-deficient HBMECs displayed a significant reduction of TEER 48 hours and 72 hours after subculture, suggesting that loss of NO leads to increased paracellular permeability (Figure 1D).

### Gene expression analysis in ASL-deficient HBMECs.

In our previous studies, we performed RNA sequencing (RNA-Seq) in ASL-deficient HAECs to investigate how loss of ASL affects human ECs on the molecular level (23). From this analysis, we identified *CLDN1* as one of the top upregulated genes in ASL-deficient HAECs. This is interesting as *CLDN1* is rarely expressed in normal BMECs but has been previously shown to be highly expressed in leaky BBB (24). We also found a significant downregulation of other BBB-associated genes, such as *CLDN5* and *CDH5* (Supplemental Table 1; supplemental material available online with this article; https://doi.org/10.1172/jci.insight.168475DS1). However, some HBMEC-specific markers, such as occludin (*OCLN*) and tight junction protein 1 (*TJP1*), were undetectable in HAECs. To further verify these findings in HBMECs, we performed quantitative reverse transcription PCR (qRT-PCR) to analyze mRNA expression of selected BBB-associated markers in HBMECs 2 days after siRNA-mediated ASL knockdown. Consistent with our RNA-Seq data in HAECs, loss of *ASL* in HBMECs resulted in a significant upregulation of *CLDN1* mRNA expression and a significant downregulation of *CLDN5*, *CDH5*, *OCLN*, and *TJP1* mRNA expression (Figure 1E). We performed Western blot to further analyze the expression of these genes on the protein level. Consistent with qRT-PCT results, CLDN1 protein expression was significantly upregulated while CLDN5 and VE-Cadherin/CD144, which is encoded by the *CDH5* gene, were significantly downregulated compared with controls (Figure 1, F and G). We did not see a statistically significant difference in OCLN protein expression. Therefore, these findings suggest that loss of ASL-dependent NO production in HBMECs leads to dysregulation of BBB-associated genes, marked by an increased expression of CLDN1 and a decreased expression of CLDN5.

### Effects of NO donor on ASL-deficient HBMECs.

**Figure 2:**
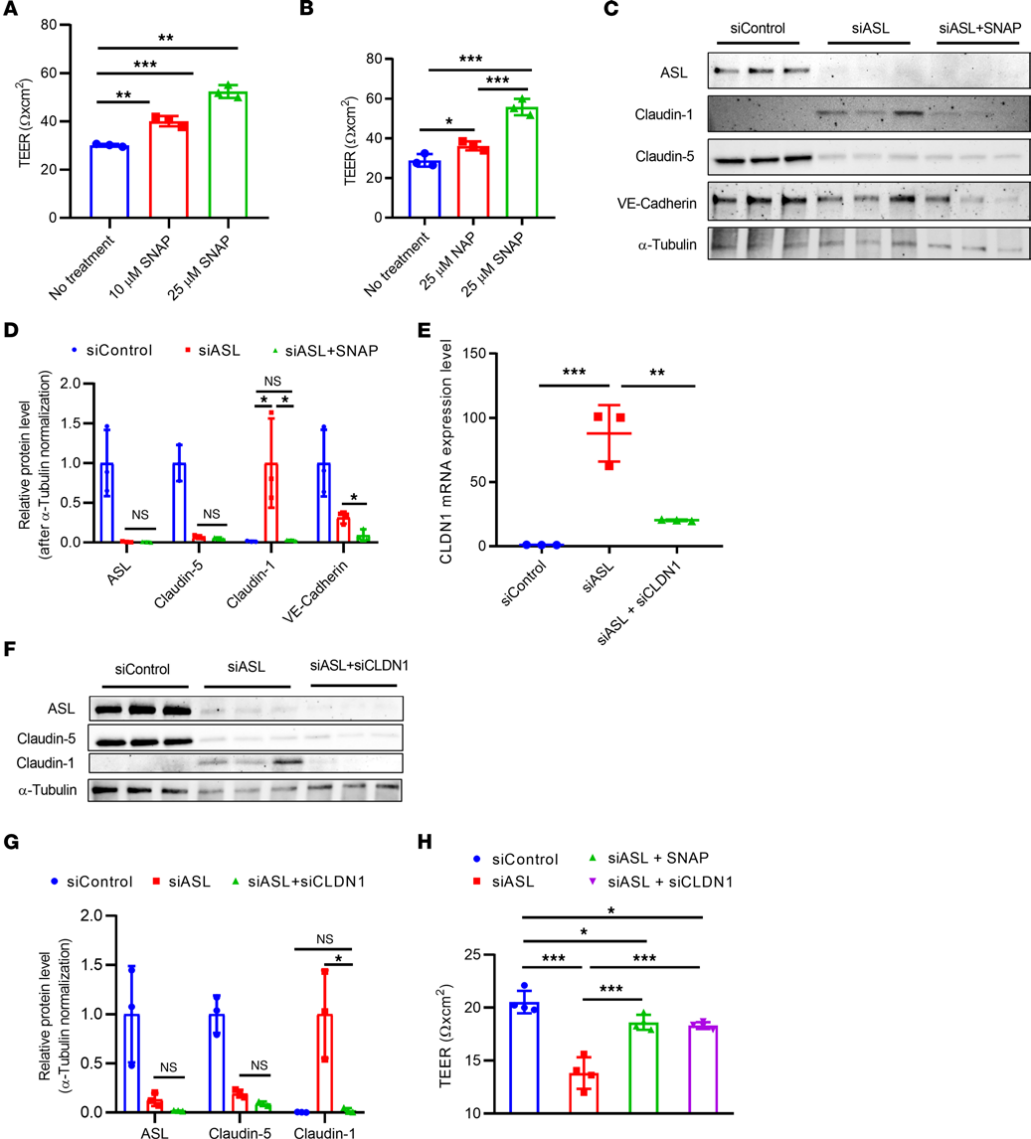
Treatment with an NO donor or Claudin-1 inhibition improved barrier integrity in ASL-deficient HBMECs. (**A**) TEER measurement in HBMECs treated with 0, 10, or 25 μM of *S*-nitroso-*N*-acetylpenicillamine (SNAP), an NO donor, 48 hours (hr) after subculture (*n* = 3). (**B**) Comparison of TEER levels in HBMECs treated with 25 μM of either *N*-acetylpenicillamine (NAP) or SNAP 48 hr after subculture. SNAP or NAP was added daily, starting 24 hr after subculture (*n* = 3). (**C**) Western blot analysis and (**D**) quantification investigating the effects of SNAP (25 μM) on expression of ASL, CLDN5, CLDN1, and VE-Cadherin in ASL-deficient HBMECs (*n* = 3). SNAP was added to the treatment group 24 hr prior to protein extraction. (**E**) Relative mRNA expression of CLDN1 in HBMECs 48 hr after transfection with siControl, siASL, or both siASL and siCLDN1 (*n* = 3). (**F**) Western blot analysis and (**G**) quantification to assess ASL, CLDN5, and CLDN1 protein level in HBMECs 48 hr after siRNA transfection (*n* = 3). Protein abundance was normalized to α-Tubulin level in **D** and **G**. (**H**) TEER measurement to investigate the effects of SNAP (25 μM) or siRNA-mediated inhibition of CLDN1 on paracellular barrier integrity of ASL-deficient HBMECs. Bar graphs represent mean values while error bars represent the standard deviation. *P < 0.05, **P < 0.01, and ***P < 0.001. One-way ANOVA, multiple comparisons.

Given that loss of ASL resulted in NO deficiency in HBMECs, we further investigated whether NO supplementation could improve barrier permeability and prevent claudin imbalance in ASL-deficient HBMECs. To test this, we first examined whether supplementation with *S*-nitroso-*N*-acetylpenicillamine (SNAP), an external source of NO, affects paracellular permeability in normal HBMECs. SNAP was added 24 hours after subculture on Transwell inserts, and TEER values were measured 48 hours after subculture. We observed a dose-dependent increase of TEER values in HBMECs treated with 10 or 25 μM of SNAP (Figure 2A). Compared with *N*-acetylpenicillamine (NAP), a negative control compound of SNAP, the increase of TEER values was significantly higher in SNAP-treated cells 48 hours after subculture (Figure 2B). These findings confirm that an NO donor can modulate paracellular permeability of HBMECs. However, at the molecular level, mRNA expression of *CLDN5*, *CLDN1*, and *CDH5* in SNAP-treated HBMECs was not significantly altered by treatment with SNAP, suggesting the NO effect was not primarily on transcription (Supplemental Figure 1).

Next, we evaluated whether treatment with SNAP could alleviate the reduced TEER phenotype in ASL-deficient HBMEC. We found that TEER in ASL-deficient HBMECs was significantly increased by treatment with 25 μM of SNAP and partially restored to the normal value reached by control HBMECs (Figure 2H). We also examined the effects of SNAP treatment on protein expression. CLDN5 and VE-Cadherin protein level remained unchanged. However, SNAP dramatically reduced CLDN1 protein expression in ASL-deficient HBMECs (Figure 2, C and D). Together, these data suggest that NO donor can modulate endothelial barrier permeability and partially rescue barrier phenotypes in ASL-deficient HBMECs, and this was associated with reduction of pathological increase in CLDN1.

### Effects of CLDN1 inhibition on ASL-deficient HBMECs.

Targeting CLDN1 using either shRNA or pharmacological approach has been shown to improve BBB recovery after stroke (24). We investigated whether such an approach can also be used to potentially alleviate BBB deficits in the ASLD model. We co-transfected HBMECs with both siASL and siCLDN1 and verified by Western blot that in siCLDN1-transfected cells, CLDN1 mRNA and protein levels remained low and were comparable to the control (siControl-transfected HBMECs) (Figure 2, E–G). We also measured TEER to test the effect of *CLDN1* inhibition on BBB deficits in ASL-deficient HBMECs. *CLDN1* suppression significantly increased TEER values in ASL-deficient HBMECs and alleviated the phenotype to a level that was comparable to SNAP treatment (Figure 2H). These results further suggest *CLDN1* upregulation as a potential driver of BBB breakdown in ASL-dependent NO loss.

### In vivo assessment of BBB in a mouse model of ASLD (Asl^Neo/Neo^ mice).

**Figure 3:**
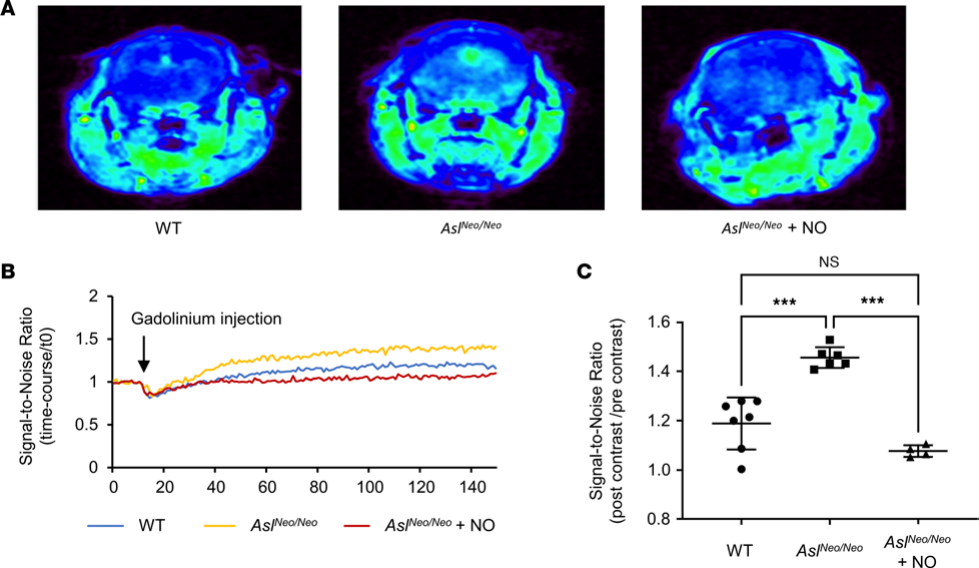
DCE-MRI analysis of BBB breakdown in *Asl^Neo/Neo^* mice. (**A**) Representative T1-weighted images acquired after injection with gadolinium contrast agent in WT, mutant (*Asl^Neo/Neo^*), and sodium nitrite–treated mutant mice (*Asl^Neo/Neo^* + NO). (**B**) The average ratio of postcontrast signal intensity to *t*0 signal intensity over the course of experiment. The entire DCE experiment took approximately 33 minutes with 150 data points collected over this time frame. Magnevist was injected 2 minutes into the scan (at data point 10 out of 150). (**C**) Signal-to-noise ratio (postcontrast/precontrast average signal intensity) in WT, mutant, and sodium nitrite–treated mutant mice. WT: *n* = 7 mice. *Asl^Neo/Neo^*: *n* = 6 mice. *Asl^Neo/Neo^* + NO: *n* = 4 mice. Bar graphs represent mean values while error bars represent the standard deviation. ***P < 0.001. One-way ANOVA, multiple comparisons.

To evaluate the effects of ASLD on BBB in vivo, we utilized a hypomorphic mouse model of ASLD, i.e., B6.129S7-*Asltm1Brle*/J (*Asl^Neo/Neo^*), that has approximately 10% residual activity of ASL (8). These mice were maintained on a low-protein diet to prevent hyperammonemia and strongly phenocopy the human condition. We first performed dynamic contrast–enhanced magnetic resonance imaging (DCE-MRI) in 5-month-old *Asl^Neo/Neo^* and wild-type (WT) mice to assess the BBB. T1-weighted scans were taken prior to and after injection with a chelated gadolinium MRI contrast agent (Magnevist). We measured the signal-to-noise ratio and found a significant increase of gadolinium enhancement across the entire brain in mutant mice, suggesting BBB breakdown in ASLD (Figure 3, A–C, and Supplemental Videos 1–3).

**Figure 4:**
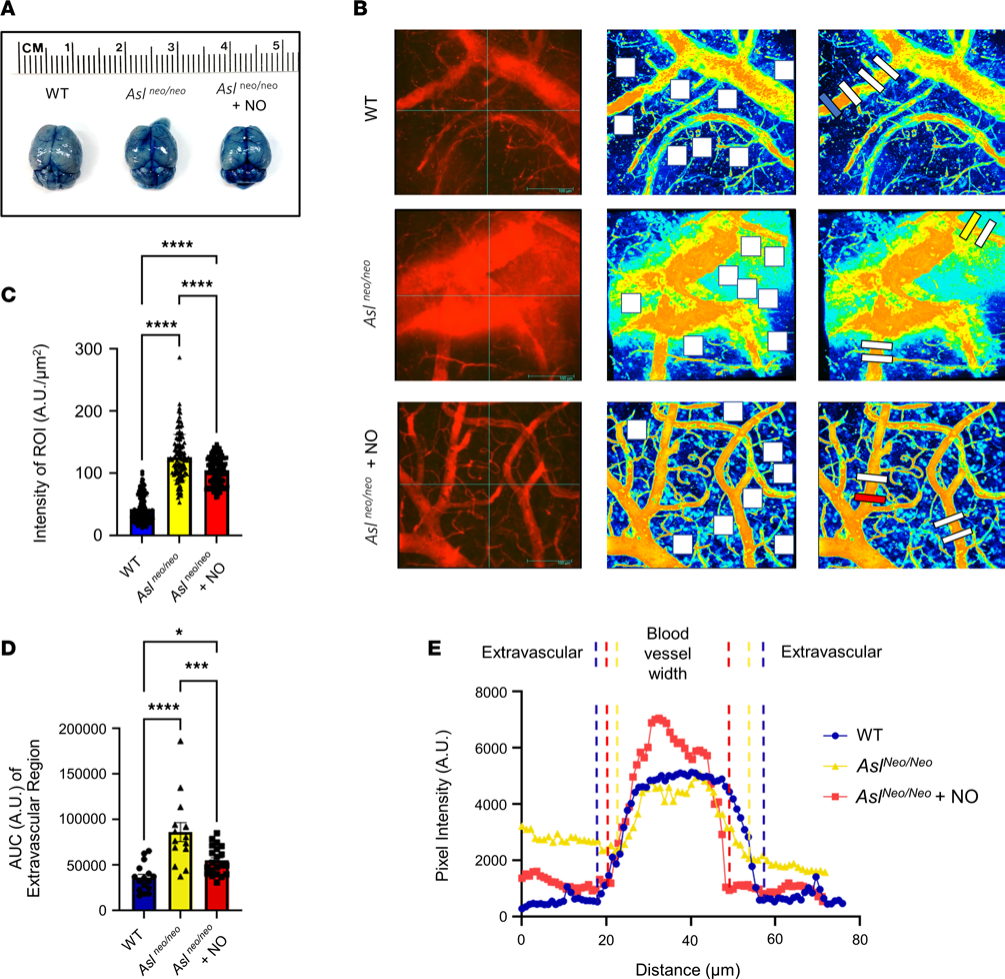
BBB breakdown in *Asl^Neo/Neo^* mice as shown by Evans blue fluorescent tracer extravasation. (**A**) Representative picture of gross distribution of Evans blue–conjugated albumin after I.V. injection in WT, mutant (*Asl^Neo/Neo^*), and sodium nitrite–treated mutant mice (*Asl^Neo/Neo^* + NO). (**B**) Two-photon images of cerebral vasculature shown as (i) maximal intensity plots and (ii) fluorescence intensity maps. Box (50 × 50 μm) plots and line (70 μm) profile of fluorescence intensity were measured at the designated locations. The box color corresponds to the genotype legend in **B**, **D**, and **E**. (**C**) Box plot ROI intensity (WT: 42.3 ± 1.9 A.U./μm^2^. *Asl^Neo/Neo^*: 125.4 ± 3.4 A.U./μm^2^. *Asl^Neo/Neo^* + NO: 104.1 ± 1.9 A.U./μm^2^.) WT: *n* = 4 mice, 3–6 images per mouse, 6–8 ROIs per image. Total 123 ROI determinations. *Asl^Neo/Neo^*: *n* = 4 mice, 3–5 images per mouse, 6–8 ROIs per image. Total 116 ROI determinations. *Asl^Neo/Neo^* + NO: *n* = 5 mice, 3–5 images per mouse, 6–8 ROIs per image. Total 134 ROI determinations. Mean ± SE. (**D**) Line profile of fluorescence intensity. Extravascular fluorescence levels were estimated by the area under the curves of the pixel intensity by subtracting that of blood vessel. (**E**) The extravascular AUC. (WT: 36,179 ± 3,836 A.U. *Asl^Neo/Neo^*: 86,453 ± 10,419 A.U. *Asl^Neo/Neo^* + NO: 55,036 ± 3,377 A.U.) Line profiles were presented as the pixel intensity. WT: *n* = 4 mice, 4-line profiles per image, 16 image determinations. *Asl^Neo/Neo^*: *n* = 4 mice, 4-line profiles per image, 21 image determinations. *Asl^Neo/Neo^* + NO: *n* = 5 mice, 4-line profiles per image, 14 image determinations. Bar graphs represent mean values while error bars represent the SE. *P < 0.05, ***P < 0.001, and ****P < 0.0001. One-way ANOVA followed by Newman-Keuls test. I.V., intravenous.

To further investigate the BBB phenotypes, we injected the same set of mice 1 month later (now 6-month-old mice) with a fluorescent tracer intravenously. Brain tissues were collected 2 minutes after injection with Evans blue–conjugated albumin. Gross distribution of Evans blue showed evidence of BBB breakdown in *Asl^Neo/Neo^* mice (Figure 4A). To further visualize and quantify the degree of BBB leakage, we performed 2-photon imaging. Representative images were taken and used to measure Evans blue extravasation (Figure 4B and Supplemental Figure 2). Regions of interest (ROIs) were randomly selected around the proximal area of the blood vessels, and the fluorescence signal intensity was quantified by using the area average for the box plots (Figure 4C) and the point-to-point function for line plots (Figure 4, D and E). Consistent with our MRI data, we found evidence of BBB breakdown in *Asl^Neo/Neo^* mice as shown by a significant increase of Evans blue dye extravasation (Figure 4D).

To test the NO dependence of the BBB dysfunction in ASLD, we also treated *Asl^Neo/Neo^* mice with sodium nitrite, an NO supplement, and discovered that BBB leakage was significantly alleviated as independently assessed by DCE-MRI (Figure 3, A–C) and Evans blue assay (Figure 4, A–E). Together, these in vivo data support our in vitro findings and suggest that ASL-mediated NO synthesis is required for proper maintenance of the BBB.

### Assessment of BBB in patients with ASLD.

**Figure 5:**
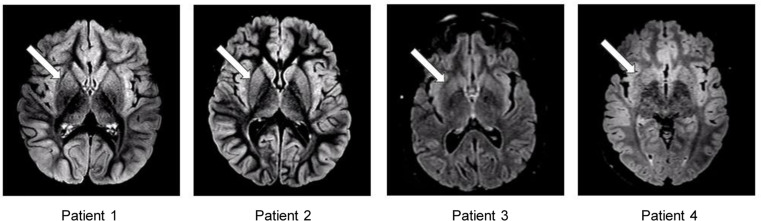
MRI analysis of BBB breakdown in individuals with ASLD. Comparison of neuroimaging findings among patients with ASLD. Abnormal hyperintensities are best seen on axial T2 FLAIR MRI for patient 1, patient 2, patient 3, and patient 4. Bilateral hyperintensities can be seen in the putamen, globus pallidus, heads of the caudate nuclei, and insular cortices. A gradation pattern (anterior to posterior) of hyperintensity can be seen in the bilateral putamen (arrow).

To assess the potential correlate of BBB dysfunction in humans with ASLD, we performed a retrospective review of individuals with ASLD who were evaluated in the metabolic clinic at Texas Children’s Hospital and had existing brain MRIs (*n* = 4). Of 4 individuals in this convenience sample, 2 were male and 2 were female. Ages, at the time of MRI, ranged from 3 years, 9 months, to 33 years (Supplemental Table 2). All MRIs were obtained for clinical indications. Although they were not specific for increased BBB permeability, we identified several abnormalities across all 4 patients (Figure 5). We observed increased hyperintensities on T2 fluid-attenuated inversion recovery (FLAIR) images localized to the bilateral putamen, globus pallidus, and heads of the caudate nuclei. We also observed hyperintensities in the bilateral putamen with a gradation pattern showing greater intensity in the anterior putamen fading into the posterior (Figure 5).

## Discussion

While NO has been previously studied in the context of the BBB (25), the dissection of such mechanisms has been hindered by complexities including multiple sources of NO production (i.e., NOS-independent and NOS-dependent), redundant enzymatic machinery for cell-autonomous production, and the promiscuity of small molecules used to inhibit or induce NO signaling. In the mouse model of experimental autoimmune encephalomyelitis, NO was shown to play both aggravating and protective roles. *eNOS*^–/–^ mice display delayed onset of BBB breakdown but also worsened recovery (26). In the mouse model of epileptogenesis, the BBB is protected in *eNOS^−/−^* and *nNOS^−/−^* mice but not in *iNOS*^−/−^ mice after kainic acid injection (27). In the mouse model of infection-induced neuroinflammation, *iNOS*^−/−^ mice also have increased BBB permeability (20). Furthermore, studies using small molecules in in vitro cell culture and in vivo animal models also show conflicting results. Treatment with *NG*-monomethyl-l-arginine acetate (an NO inhibitor) in rats did not alter BBB permeability (28) while NO donors, such as SNAP or SIN-1, could cause an increase in permeability (22). Treatment with SNAP in a cell culture model of BBB, however, demonstrated the protective effect of NO on BBB (29). Another study using a mouse model of transient focal cerebral ischemia showed that different NOS inhibitors have differential effects on the BBB (30).

Here, we have attempted to overcome the challenges of studying NO production by focusing on the specific source of all NOS-dependent NO, i.e., the pool of arginine that is produced in cell-specific fashion. NO can be produced by NOS-dependent mechanisms via conversion of arginine substrate to citrulline product. Citrulline can be subsequently recycled back into arginine via 2 ubiquitous enzymes: argininosuccinate synthetase 1, which converts citrulline and aspartate into ASA, and ASL, which cleaves ASA into arginine substrate and fumarate. Because ASL is the sole enzyme responsible for intracellular production of arginine in mammals and is structurally required for channeling of extracellular arginine for NO production, cells, mice, and humans with ASLD exhibit cell-autonomous deficiency of NO production (8, 11, 23). Loss of ASL abolishes all NOS-dependent NO production, and thus, ASLD serves as a model to study the roles of NO in regulating the BBB.

We demonstrated that loss of ASL-mediated NO synthesis in HBMECs led to increased cell permeability and dysregulated expression of junctional proteins, including claudin-1 and claudin-5. Moreover, loss of NO in the hypomorphic mouse model of ASLD in vivo led to BBB breakdown. Our findings also provided a potentially novel explanation for the pathogenesis of neurocognitive dysfunction in ASLD. We showed that beyond its well-known roles in regulating vascular tone, NO regulates the paracellular permeability of CNS-derived ECs and protects against BBB breakdown in this model. We also performed a retrospective study of MRI data from 4 individuals with ASLD to assess whether the BBB is compromised in these patients. We identified signal abnormalities that were consistent across all 4 patients and marked by increased hyperintensities on T2 FLAIR images localized to the bilateral putamen, globus pallidus, and heads of the caudate nuclei. Signal abnormalities of the posterior-lateral aspect of the putamen and bilateral insular cortex have been reported previously in 2 siblings with arginase deficiency, a UCD distal to ASLD in the metabolic pathway (31). Additionally, in a cohort of 56 individuals with ASLD, of the 21 patients who had brain MRIs, 4 had white matter hyperintensities on T2 sequences with at least 1 patient also having abnormalities in the bilateral heads of the caudate and posterior putamina (3). Enhancing lesions can be indicative of increased BBB permeability as intravascular contrast material extravasates through the BBB; however, these enhancing lesions are also nonspecific, and more specialized imaging modalities are needed to accurately measure BBB permeability in vivo (32). These CNS findings, and those observed in our patients, illustrate the need for further specialized studies to assess BBB permeability in individuals with ASLD, especially as NO supplementation is being studied as an intervention for this condition (ClinicalTrials.gov NCT03064048).

Mechanistically, our findings suggest that these phenotypes are likely driven by the imbalance of claudins, as shown by significant downregulation of claudin-5 and upregulation of claudin-1. Claudin-5 is the most enriched tight junction protein in CNS-derived ECs and is known for its crucial roles in maintaining BBB integrity. Trans-interaction of claudin-5 strands provides a mechanical link between individual BMECs and regulates the diffusion of molecules across the BBB. *Cldn5* global knockout mice display aberrant BBB permeability and die within 10 hours of birth (33). Claudin-5 dysfunction has also been implicated in many neurological disorders, including Alzheimer’s disease, multiple sclerosis, stroke, epilepsy, and schizophrenia (34). Claudin-1 functions in the BBB are not yet well studied, especially since claudin-1 is not highly expressed at the BBB under normal physiological conditions. Although *Cldn1* global knockout mice also die shortly after birth, this is mainly caused by epidermal barrier defect and not BBB breakdown (35). Interestingly, induction of claudin-1 has been shown to drive BBB leakage in chronic stroke (24). Similar to our findings, Sladojevic et al. also found a significant upregulation of claudin-1 and downregulation of claudin-5 in poststroke conditions in both mice and patients (24). They found that the newly synthesized claudin-1 established stable interactions with ZO-1 and was incorporated into the tight junction complex. Claudin-1 strand formation interrupted trans-interaction of claudin-5 and claudin-5/ZO-1 interaction, altering claudin-5 incorporation into the tight junction complex. They also showed that blocking claudin-1 with a peptide (C1C2) could improve BBB recovery in vitro and in in vivo mouse models. In our study, we found that knocking down *CLDN1* in ASL-deficient HBMECs also improved BBB integrity. These findings suggest that abnormal induction of claudin-1 is one of the main drivers of BBB breakdown in ASL-dependent NO-deficient HBMECs.

While genetic depletion of NO signaling resulted in altered expression of both claudin-5 and claudin-1, treatment with SNAP did not affect the mRNA expression of either claudin. However, SNAP treatment substantially reduced claudin-1 protein level in ASL-deficient HBMECs. These results suggest that NO exerts its effects on claudin-1 through posttranslational protein modification. One potential mechanism is protein *S*-nitrosylation, which may affect protein stability (36). Whether claudin-1 is indeed a direct target of protein *S*-nitrosylation warrants further investigation, though other regulatory mechanisms are likely to contribute. Nevertheless, our study further highlights the potential benefit of NO supplementation for patients with ASLD, an approach currently under investigation, which adds to the potential implications of this work. Previous studies have shown that NO supplementation could improve hypertension as well as motor and cognitive deficits in different mouse models of ASLD (8, 11–13, 23). In this study, using both in vitro and in vivo models, we demonstrated that treatment with SNAP or sodium nitrate alleviated the BBB breakdown. Thus, it provides another mechanism on how NO supplementation can potentially treat neurocognitive dysfunctions in ASLD. Last, our study also highlights claudin-1 as a potential therapeutic target for preventing BBB breakdown in ASLD.

## Methods

### EC culture and siRNA experiments.

HBMECs (Cell Systems, ACBRI 376) were cultured in EGM2-MV BulletKit Medium (Lonza). The passage number of HBMECs used for the experiments was between 10 and 13. To perform siRNA-mediated knockdown of ASL, HBMECs were plated overnight in 12-well plates at 6 × 10^5^ cells/well density and transfected with 24 pmol siRNA and 4 μL Lipofectamine RNAiMAX reagent (Thermo Fisher Scientific) per well. For double-knockdown experiments, 12 pmol of siASL was mixed with either 12 pmol of siCLDN1 or nontargeting siControl. siRNAs targeting human *ASL* or *CLDN1* were obtained from Thermo Fisher Scientific (Silencer Select; s1669 for siASL, s17315 for siCLDN1). siControl was also obtained from Thermo Fisher Scientific (Silencer Select Negative Control No. 1 siRNA, 4390843) and used as a control in all experiments. RNA was extracted 48 hours posttransfection to assess knockdown efficiency.

To test the effects of NO donor, SNAP (Cayman Chemical) was added to HBMECs at 10 or 25 μM concentration. NAP (Cayman Chemical) was used as the negative control (Figure 2B) and added at a similar concentration (25 μM). For the in vitro NO rescue experiments (Figure 2, C, D, and H), SNAP was added 48 hours posttransfection (24 hours after cells were plated onto the Transwell inserts).

### RNA isolation and qRT-PCR.

Total RNA was extracted from HBMECs using Direct-zol RNA MiniPrep (Zymo Research) with on-column DNase digestion. First-strand cDNA was synthesized using SuperScript III First-Strand Synthesis System (Thermo Fisher Scientific). qRT-PCR was performed on LightCycler instruments (Roche) using SYBR Green I Master reagents (Roche) according to the manufacturer’s recommendations. Relative mRNA expression was normalized to a reference gene (*GAPDH*).

### TEER measurement.

To measure TEER, cells were plated into semipermeable Transwell inserts (Corning, 3470) coated with 4:1:5 collagen IV (MilliporeSigma, C5533)/fibronectin (MilliporeSigma, F0895)/water coating solution on the luminal surface, for at least 4 hours at 37°C (37). The cells were cultured for 48 hours at 37°C, and ECs were seeded in the luminal compartment at a density of 1.5 × 10^5^ cells/cm^2^ in EGM2-MV. All TEER readings were measured using STX2 chopstick electrodes with an EVOM2 volt/ohm meter (World Precision Instruments). To calculate TEER, the measured resistance (average reading normalized to cell-free inserts) was multiplied by the surface area of the Transwell inserts (in cm^2^).

### NO and cGMP measurement.

NO and cGMP were quantified as previously described (23). DAF-FM Diacetate (Thermo Fisher Scientific) was used to measure relative NO concentration. cGMP concentration was measured using competitive ELISA (Enzo), and the assay was performed as per the instructions in the product manual.

### Western blot.

To extract protein from HBMECs, cells were washed with 1× PBS and lysed in CelLytic MT Cell Lysis Reagent (MilliporeSigma) supplemented with Protease Inhibitor (Roche, 11836170001). A standard protocol was used for Western blotting. Briefly, samples were resolved in an SDS polyacrylamide gel, blotted onto a 0.45 μm PVDF membrane (Thermo Fisher Scientific), and incubated overnight at 4°C with the following primary antibodies: ASL antibody (Abcam, ab201025), claudin-1 antibody (Thermo Fisher Scientific, 51-9000), claudin-5 antibody (Thermo Fisher Scientific, 35-2500), occludin antibody (Thermo Fisher Scientific, 33-1500), VE-Cadherin antibody (R&D Systems, AF938), and α-Tubulin antibody (MilliporeSigma, T5168). The membranes were incubated with the corresponding secondary antibodies on the following day. Western blot images were taken using the ChemiDoc MP Imaging System (Bio-Rad) and further analyzed using Bio-Rad Image Lab Software.

### Mice.

Generation of *Asl^Neo/Neo^* mice was previously described (8). The mouse colony was housed in the Baylor College of Medicine Transgenic Mouse Facility. WT and *Asl^Neo/Neo^* mice were maintained on low-protein diet (8% protein, Envigo, TD.93033) upon weaning (3 weeks of age). Protein-adjusted diets were provided to prevent hyperammonemia and improve the survivability of *Asl^Neo/Neo^* mice. Both sexes of mice were used for the experiments. For NO supplementation, mice were treated with sodium nitrite (10 mg/kg/d) mixed in the drinking water. Sodium nitrite was provided to the breeding cage before delivery and throughout development until the day of the experiments. The same mice were used for both fluorescent tracer and MRI studies.

### MRI and analysis in mice.

All MRI was performed using a 9.4 T Bruker AV NEO MRI with Paravision 360 software. Mice were anesthetized with isoflurane and transferred supine to the animal imaging holder, then transferred to the imaging instrument. A pressure-sensitive pillow was placed on the abdomen to constantly monitor respiration (Small Animal Instruments). The body temperature of the animal was constantly monitored using a rectal probe and maintained at 37°C using an air heater (Small Animal Instruments). Briefly, for DCE studies, an intravenous line was placed in the tail vein of a mouse prior to placement within the magnet. A localizer scan was used to center the mouse. A 3-dimensional spoiled gradient recalled echo sequence with a fixed flip angle of 16° over 175 repetitions was used to obtain the dynamic data. For the spoiled gradient recalled echo sequence, the repetition time = 6 ms, echo time = 1.93 ms, and acquisition matrix = 128 × 128 × 16 over a 3.0 cm × 3.0 cm × 2.5 cm field of view yielded a voxel resolution of 0.234 mm × 0.234 mm × 1.562 mm. Two minutes into the dynamic acquisition, the contrast agent was injected as a bolus via the tail vein line attached to a syringe pump. Magnevist was administered intravenously at an initial volume of 200 μL per 30 g body weight. We chose this injection volume because it is considered safe based on the blood volume of a mouse according to the Baylor College of Medicine veterinary care staff. DCE-MRI was performed in WT and *Asl^Neo/Neo^* mice. MRI scans were analyzed with OsiriX software.

### Fluorescent tracer injection, imaging, and analysis.

Evans blue (3% *w/v*) was dissolved in saline containing the equal amount of bovine serum albumin (3% *w/v*). The unbound Evans blue was removed through centrifugation at 12,000*g* for 15 minutes at room temperature with the spin column (MilliporeSigma, molecular weight cutoff: 100 kDa). Evans blue was reconstituted at its original volume with saline, and then the solution was immediately used for the experiment. Mice under isoflurane anesthesia were injected with 200 μL of the solution in the tail or jugular vein. Mice were sacrificed 2 minutes after the injection by cardiac puncture via heparin-coated syringes to obtain blood. Brain tissue was immediately dissected and placed on ice. Plasma was isolated via centrifugation at 2,350*g* for 15 minutes at room temperature and stored at –80°C for further processing.

Brain tissue imaging was performed with a custom-made 2-photon microscope system (Bruker Nano, Ultima investigator) equipped with a femtosecond pulse laser module (Spectra-Physics, Deep See). The excitation wavelength was set at 810 nm, and the emission was acquired through GaAsP detectors, which were controlled by InSight software (version 1.01.60) and Prairie View software (version 5.5), respectively. Imaging parameters used were imaging pockels at 50, detector sensitivity at 750, and image resolution at 512 × 512 pixels. *Z*-stack images were obtained 200–250 μm from the cortical surface at a 1 μm increment per image. Each imaging plane was averaged by 16 frame exposures to normalize the background.

The maximal intensity plot was served for the quantification of image. Fluorescence intensity mapping for each image was generated with Arivis Vision 4D (version 3.3) to determine the outer edge of the vascular structures. The extravasation of Evans blue was determined as the fluorescent signals out of the blood vessels. The signal intensity of the ROI was quantified by using the point-to-point function for line plots and the area average for the box plots. The ROIs were randomly determined at the proximal area of the blood vessels in the right brain hemisphere. The pixel intensity in line plots was analyzed across the vessel ±20 μm perpendicular to the vessel borders. The box plot pixel intensity was quantified and averaged for each ROI from 60 × 60 μm region. The ROIs were further averaged with *n* = 4 determinations per image for the comparisons among cohorts. Each brain sample was randomly imaged at 4–5 different locations in the parieto-occipital regions on the right hemisphere. The image analysis was performed in a blinded manner.

### Human study.

This was a single-center, retrospective study that was approved by the Institutional Review Board at Baylor College of Medicine. Inclusion criteria were patients with ASLD seen at the Texas Children’s Hospital metabolic clinic between January 1, 1995, and December 31, 2020, who had also had brain MRI done as part of their care. A total of 5 patients were identified and 1 was excluded as imaging for this patient was not available in our system. Brain MRIs were reviewed by a single pediatric neuroradiologist. Charts were reviewed to collect clinical information summarized in Supplemental Table 2. Patient 1 was a developmentally appropriate female who underwent liver transplant at age 4 years due to recurrent hyperammonemic episodes. Her MRI was obtained prior to transplant at 3 years, 9 months. Patient 2 was a 10-year-old male with intellectual disability (ID), spasticity, and recurrent hyperammonemic episodes. He was treated with nitrogen scavengers, supplemental arginine, and protein-restricted diet, to 100% of his daily recommended intake (DRI). Patient 3 was a 33-year-old woman. She had a history of seizures, spasticity, and ID. She has never had documented hyperammonemia despite an unrestricted diet and no nitrogen scavengers or arginine treatment. Patient 4 was a 21-year-old man with a history of hypertension and ID. He too has never had hyperammonemia; he does not require nitrogen scavengers but does restrict protein to 50% of DRI and takes supplemental arginine.

### Statistics.

Statistically significant differences between 2 groups were determined by 2-tailed Student’s *t* test. Comparisons between multiple groups were performed by 1-way ANOVA followed by Tukey’s multiple-comparison or Newman-Keuls test. A *P* value less than 0.05 was considered significant.

### Study approval.

The human study was performed in accordance with a research protocol (H-48670) that was approved by the Institutional Review Board of Baylor College of Medicine. All research data were coded and stripped of personal health identifiers. All experimental procedures were approved by the Baylor College of Medicine Institutional Animal Care and Use Committee and Center for Comparative Medicine (Baylor College of Medicine Animal Protocol AN-4822, AN-1506, and AN-6718).

### Data availability.

The data supporting the findings reported in this study are available in the Supporting Data Values file. The raw RNA-Seq data were previously published (23) and deposited in the National Center for Biotechnology Information Sequence Read Archive database under the accession code SRP152883.

## Author contributions

JK designed the experiments, performed most of the experiments, and wrote the manuscript. UP assisted with mouse experiments and statistical analysis. MMJ assisted with in vitro studies in HBMECs. AU and HPT performed the fluorescent tracer injection, imaging, and analysis. RGP assisted with MRI and analysis in mice. JDO, JVH, SMA, LCB, and SCSN performed the MRI analysis in individuals with ASLD. ZJ assisted with manuscript preparation and wrote the manuscript. BL supervised the project and wrote the manuscript.

## Supplementary Material

Supplemental data

Supplemental video 1

Supplemental video 2

Supplemental video 3

Supporting data values

## Version 1 07/25/2023

In-Press Preview

## Version 2 09/08/2023

Electronic publication
