# Supplementary material for: Argininosuccinate lyase deficiency causes blood-brain barrier disruption via nitric oxide–mediated dysregulation of claudin expression
Source: JCI Insight. 2023 Sep 8;8(17):e168475. doi: 10.1172/jci.insight.168475 (PMC10544197; doi:10.1172/jci.insight.168475)
Supplement: Supplemental data [file jciinsight-8-168475-s163.pdf]

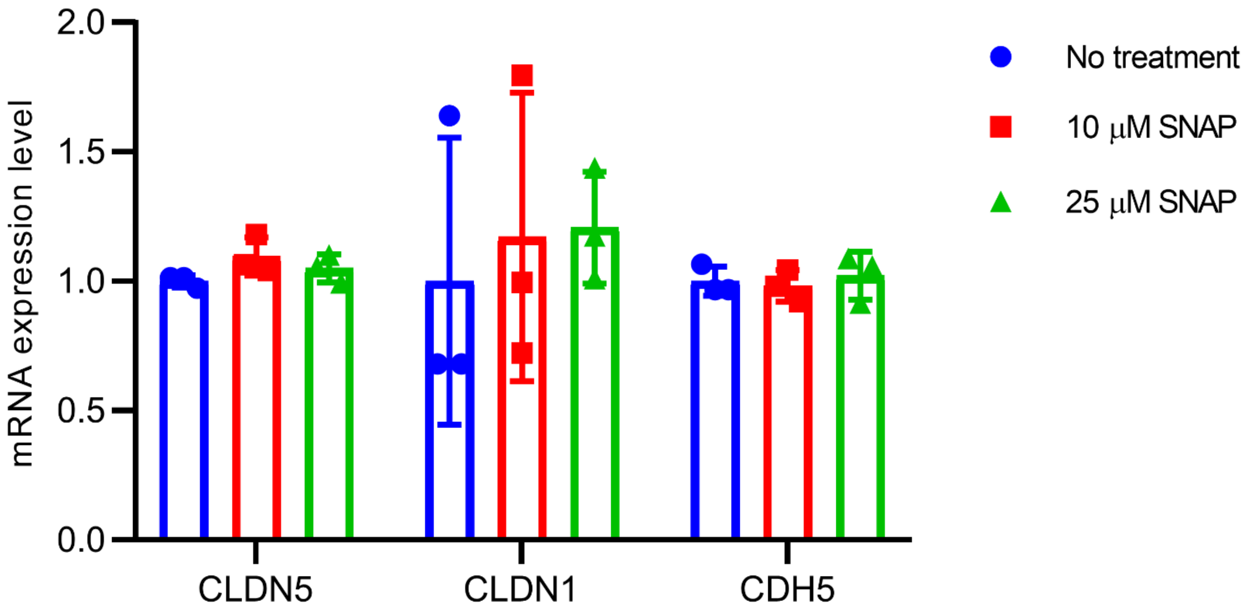

Supplementary Figure 1. Effects of NO Donor on mRNA expression of BBB genes
mRNA expression of *CLDN5*, *CLDN1*, and *CDH5* in HBMECs treated with 0, 10, or 25 µM of S-nitroso-N-acetylpenicillamine (SNAP), an NO donor (n = 3). Bar graphs represent mean values while error bars represent the standard deviation. One-way ANOVA, multiple comparisons.

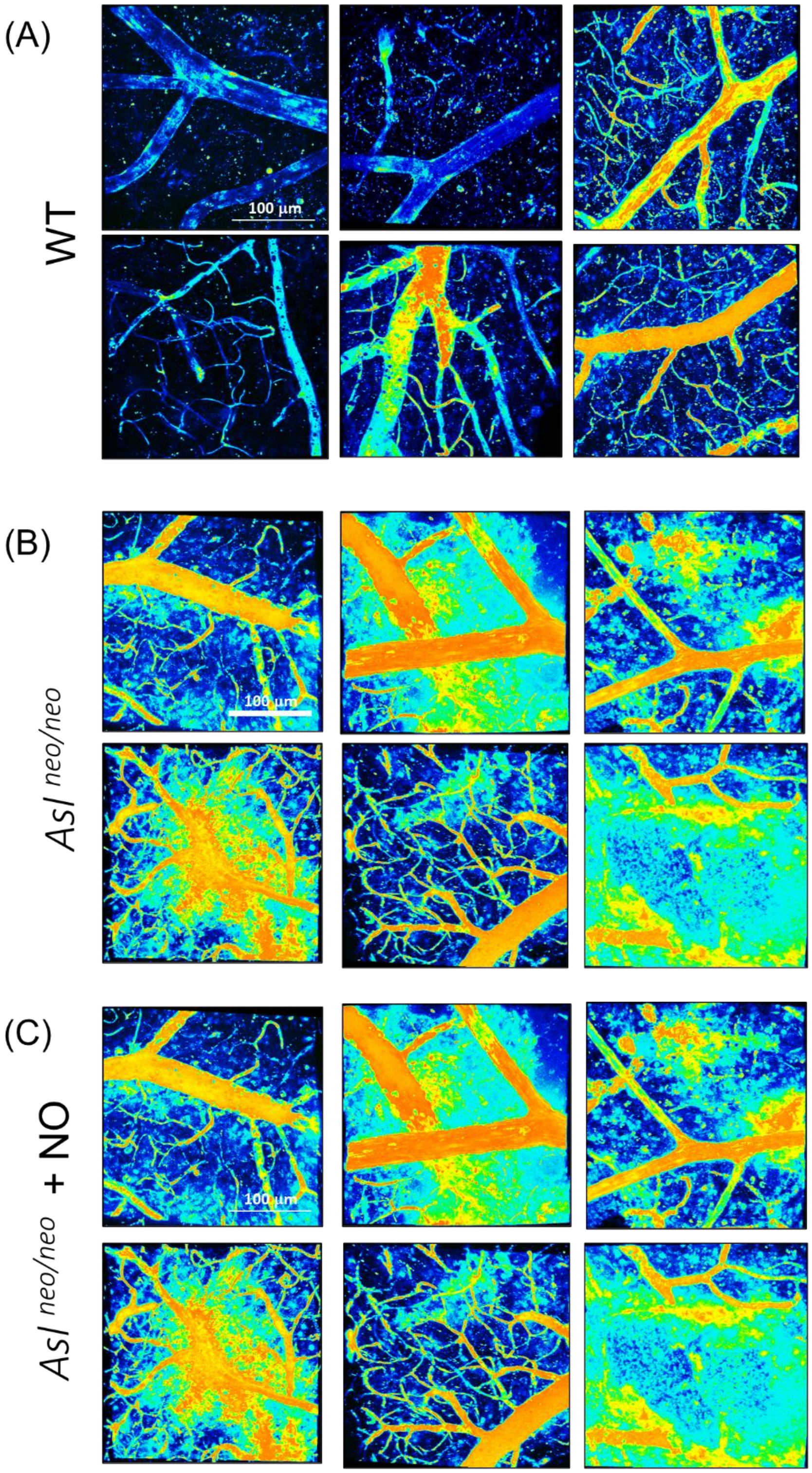

Supplementary Figure 2. Additional images to supplement Figure 4. Two-photon images of cerebral vasculature in (A) WT, (B) mutant (*Asl*$^{Neo/Neo}$), and (C) sodium nitrite-treated mutant mice (*Asl*$^{Neo/Neo}$+ NO) after Evans blue-fluorescent tracer injection.

Gene		FPKM		log2(fold change)
		siControl	siASL	
ASL	Argininosuccinate lyase	15.06	0.52	-4.86
CLDN1	Claudin-1	0.22	5.10	4.52
CLDN5	Claudin-5	148.30	45.59	-1.70
CDH5	Cadherin-5	668.29	177.28	-1.91
ABCB1	Multidrug resistance protein 1 (P-glycoprotein 1)	1.47	1.07	-0.45
HSP90AA1	Heat shock protein HSP 90-alpha	369.38	437.26	0.24

Supplementary Table 1. Effects of ASL deficiency on expression of BBB-associated genes in human aortic endothelial cells (HAECs).

**Patient**	**1**	**2**	**3**	**4**
Sex	Female	Male	Female	Male
Age of diagnosis	Birth	Birth	-	3.5 years
Diagnosis	Newborn screen, biochemical testing	Clinical symptoms, biochemical testing	-	Clinical symptoms, biochemical testing
Genotype (NM_000048.3)	c.1060C>T (p.Gln354Ter), homozygous	c.765dupG (p.Met256Aspfs*79), c.1193C>A (p.Ala398Asp)	-	c.557G>A (p.Arg186Gln), c.857A>G (p.Gln286Arg)
**Patient demographics at the time of MRI**				
Age	3 y 9 mo	10 y	33 y	21 y
Weight in kg (Z-score)	16.5 (0.53)	30.3 (-0.59)	55.7	88.1
Height in cm (Z-score)	102.4 (0.72)	122 (-2.72)	155.2	185.1
**Treatment at the time of MRI**				
Liver transplant (age)	Yes (4 years old)	No	No	No
Arginine dose (g/m^2)	2.2	1.8	10	1.87
Nitrogen scavengers	Yes	Yes	No	No
Protein restriction (% DRI)	No (100%)	No (100%)	No (Not counting protein)	Yes (50%)
**Phenotype**				
Highest recorded ammonia in medical record (µmol/L)	350	216	<9	27
Number of hyperammonemic episodes	6	5	0	0
Intellectual Disability	No	Yes	Yes	Yes
Seizures	No	No	Yes	No
Spasticity	No	Yes	Yes	No
Hypertension	No	No	No	Yes

"-" datum not available

Supplementary Table 2. Demographic data of the patients with ASLD evaluated in the study.
